# Advancing Grain Legumes Domestication and Evolution Studies with Genomics

**DOI:** 10.1093/pcp/pcac062

**Published:** 2022-05-10

**Authors:** Hailin Zhang, Martin Mascher, Shahal Abbo, Murukarthick Jayakodi

**Affiliations:** Leibniz Institute of Plant Genetics and Crop Plant Research (IPK), Corrensstraße 3, Gatersleben, Seeland 06466, Germany; Leibniz Institute of Plant Genetics and Crop Plant Research (IPK), Corrensstraße 3, Gatersleben, Seeland 06466, Germany; German Centre for Integrative Biodiversity Research (iDiv) Halle-Jena-Leipzig, Puschstraße 4, Leipzig 04103, Germany; The Levi Eshkol School of Agriculture, The Hebrew University of Jerusalem, POB 12, Rehovot 7610001, Israel; Leibniz Institute of Plant Genetics and Crop Plant Research (IPK), Corrensstraße 3, Gatersleben, Seeland 06466, Germany

**Keywords:** Anti-nutrition, domestication, domestication syndrome, genomics, grain legume, nutrition, symbiosis

## Abstract

Grain legumes were domesticated in parallel with cereals in several regions of the world and formed the economic basis of early farming cultures. Since then, legumes have played a vital role in human and animal diets and in fostering agrobiodiversity. Increasing grain legume cultivation will be crucial to safeguard nutritional security and the resilience of agricultural ecosystems across the globe. A better understanding of the molecular underpinnings of domestication and crop evolution of grain legumes may be translated into practical approaches in modern breeding programs to stabilize yield, which is threatened by evolving pathogens and changing climates. During recent decades, domestication research in all crops has greatly benefited from the fast progress in genomic technologies. Yet still, many questions surrounding the domestication and diversification of legumes remain unanswered. In this review, we assess the potential of genomic approaches in grain legume research. We describe the centers of origin and the crucial domestication traits of grain legumes. In addition, we survey the effect of domestication on both above-ground and below-ground traits that have economic importance. Finally, we discuss open questions in grain legume domestication and diversification and outline how to bridge the gap between the preservation of historic crop diversity and their utilization in modern plant breeding.

## Introduction

Legumes (Fabaceae) are economically the most important to global agriculture after the grasses (Poaceae) (Smýkal et al. 2015). The grain legumes are members of the family Fabaceae harvested as dry seeds, while other members are grown for vegetables, forage and other uses. Based on the growth seasons, grain legumes are also referred to as cool-season legumes such as pea (*Pisum sativum*), lentil (*Lens culinaris*) and faba bean (*Vicia faba*) and warm-season legumes like soybean (*Glycine max*), common bean (*Phaseolus vulgaris*), peanut (*Arachis hypogaea*) and *Vigna* spp. Since grain legumes are rich in protein, dietary fiber, vitamins and minerals (Mudryj et al. 2014), they have been used for human nutrition and livestock feeding for millennia. Legumes fix atmospheric nitrogen through symbiotic rhizobial bacteria (Oldroyd et al. 2011), reducing the need for inorganic nitrogen (N) fertilizers. In rotation systems, they can improve the yield of other crops such as cereals (Cernay et al. 2018). Against the backdrop of climate change and global food demand, increasing grain legume production is crucial to safeguard food and nutritional security without losing agro-biodiversity. Herein, plant domestication refers to the transformation of wild taxa to forms amenable to profitable human husbandry. Understanding the genetic basis of plant domestication can boost modern crop adaptation and improvement (Abbo et al. 2009, 2014). Over several decades, untangling the impact of natural selection and the evolutionary history of domesticated plants has been a major research focus of geneticists (Purugganan 2019). In the Near East, the Neolithic founder legumes include pea, lentil, bitter vetch (*Vicia ervilia*), chickpea (*Cicer arietinum*) and presumably faba bean, which were first domesticated together with the founder cereals approximately 10,500 years ago (Abbo et al. 2005, 2009, Gopher et al. 2021).

Hitherto, domestication has been studied more intensely in cereals than in legumes (Abbo et al. 2009). On the one hand, the lack of genomic resources has posed an obstacle for evolutionary research; on the other hand, some grain legumes such as bitter vetch are considered as neglected or ‘orphan’ crops and have received little attention from breeders and researchers. Classically, two complementary lines of evidence including archeological records and genetic data are routinely applied to study domestication. Archaeobotany probes plant remains from multiple archeological sites with regard to their origin in time and space to infer the timing of domestication and to discover crop dispersal routes (Caracuta et al. 2016, Mascher et al. 2016). However, the archeological record is always fragmentary, posing obstacles to the accurate interpretation of the origin and spread of domesticated crops (Kantar et al. 2017). By contrast, the genetic approach employs top-down and bottom-up methods to trace the genetic footprints of domestication and diversification using extant individuals (Ross-Ibarra et al. 2007). The top-down method uses quantitative trait locus (QTL) mapping and genome-wide association studies (GWASs), both of which require phenotypes. In addition, the bottom-up approach is to identify selection signatures by querying extant genetic variation data, which does not need phenotypes.

Over the years, increases in throughput capacity and the reduction in the cost of DNA sequencing have led to the widespread adoption of genomic approaches in domestication and crop evolution studies. In this review, we outline a workflow to study legume domestication using genomics. We then describe the key domestication traits in grain legumes and their centers of origin. We review how genomics has been employed to discover genetic determinants of domestication and diversification traits in grain legumes. Additionally, we review the effects of domestication on grain legume metabolic and symbiosis traits. Finally, we discuss the differences between grain legumes and cereals and provide future directions to study grain legume domestication and evolution and how this knowledge can be translated into improved crop varieties.

## Roadmap to Study Legume Domestication with Genomics

**Fig. 1:**
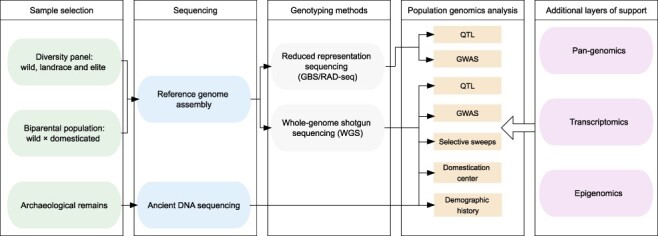
Workflow to investigate grain legume domestication using genomics and supporting layers of data from transcriptomics, pan-genomics and epigenomics.

Genomics plays an essential role in identifying genes involved in the domestication and diversification of crops (Schreiber et al. 2018). Typically, domestication studies can benefit from reference genome assembly, genome-wide genotyping of large germplasm collections comprising wild germplasm, landraces and elite genotypes and biparental mapping populations derived from wild × domesticated genotypes (**Fig. 1**). A reference genome assembly is a prerequisite for discovering molecular markers across chromosomes and performing genetics and population genomic analysis. De novo genome assembly for single or multiple domesticated and wild accessions is now realistic for many crops, owing to the cost reduction and the development of highly accurate long-read sequencing technology such as PacBio HiFi (Wenger et al. 2019). Genotyping can employ reduced representation approaches such as genotyping-by-sequencing (GBS) and whole-genome shotgun sequencing (WGS) that affords orders of magnitude more markers than GBS. Reduced representation approaches may provide sufficient resolution for a top-down approach, where biparental mapping populations or diversity panels are used for QTL mapping and GWAS, respectively. However, GBS has less power in tracking local evolutionary signatures in bottom-up approaches. In the past, it was often necessary to strike compromises between panel size and sequence coverage for WGS in crops with large genomes, i.e. to decide whether to sequence more samples at low coverage or fewer samples at high coverage. Also, gene-centric complexity reduction methods such as exome capture and RNA-seq were commonly applied. This dilemma persists in principle, but the cost decreases in recent years have made medium- to high-coverage WGS of panels comprising hundreds of diverse accessions feasible even in species with large genomes and rendered exome capture cost-ineffective as it entails much higher cost for library preparation than WGS. Advances in genotype imputation tools and improved statistical methods permit the sequencing of hundreds to thousands of genotypes at low sequence coverage (1×–5× coverage). The missing data points in the variant matrix can be imputed with computational tools such as BEAGLE (Browning et al. 2021) or IMPUTE2 (Howie et al. 2009).

We propose that population genomic studies aiming to answer key questions in crop evolution should generate WGS data for wild and domesticated (landraces and elites) cultivars. With these genomic data in hand, analyses can shed light on the crop’s center of diversity, determine whether it had one or multiple genetically independent origins, and unravel dispersal routes. The comparisons of wild and landrace populations will pinpoint the genomic regions that were selected ever since domestication until the onset of modern plant breeding; the comparisons between landrace and elite populations can expose the regions targeted by breeders in the past century. Population genomic analysis can also identify key events and trajectories in the demographic history of crops (Doebley et al. 2006). These include (but are not limited to) an approximate timing of domestication and split times of subpopulations. Various demographic models such as the site frequency spectrum in ‘fastsimcoal2’ (Excofffier et al. 2021), joint frequency spectrum in ‘dadi’ (Gutenkunst et al. 2009), pairwise sequentially Markovian coalescent model (PSMC), etc. (Terhorst et al. 2017) have been developed to infer population size history. One caveat is that PSMC and similar methods may require high sequence coverage (∼10–30×) (Nadachowska-Brzyska et al. 2016) to resolve heterozygosity. In the case of self-pollinating species, it must be noted that PSMC and similar tools were not designed with inbreeding and homozygous genomes in mind. Care must be taken when interpreting hybrids between populations: e.g., recent crop-wild hybrid derivatives (Campbell et al. 2016) may be mistaken as ancestral forms as they are expected to occupy intermediate positions between wild and domesticated forms.

Dense genomic marker data from WGS enable us to infer footprints of selection (Hermisson and Pennings 2017, Alachiotis and Pavlidis 2018). The signals most commonly sought for are selective sweeps, evolutionary events that gave rise to large blocks (hundreds of kilobases to tens of megabases) of linked polymorphisms that are fixed or segregating at high frequency (haplotype blocks) in the domesticated form. In the wild progenitor, by contrast, blocks of linked variants are shorter and more diverse, with many of them segregating at low or medium frequencies (Hermisson and Pennings 2017). Ancient DNA research, also known as archeogenomics, allow us to access directly past genetic diversity that may have been lost during evolution and to support time estimates of the domestication process and expose molecular diversity in key genes during the domestication episode or of ancient/extinct crop forms (Mascher et al. 2016). The key conceptual drawback of ancient DNA studies is the haphazard nature of their design: it requires a fair share of good luck to unearth ancient plant remains that contain enough DNA for molecular analysis. Many regions of the world, e.g., wet temperate climate zones or the tropics, are not conducive to the preservation of DNA. Even if well-preserved ancient samples are available, technical challenges need to be overcome. The level of DNA degradation is a major limitation in ancient DNA and thus the extracted DNA molecules tend to be short (<50 bp) (Sawyer et al. 2012). In addition, deamination of cytosine to uracil occurs frequently at the ends of these fragments, which impedes the analysis of sequenced fragments while also confirming the authenticity of the material (Sawyer et al. 2012). Nevertheless, efficient protocols for ancient DNA extraction and subsequent sequencing library preparation have been developed (Gamba et al. 2016, Gansauge et al. 2017). If sufficient amounts of ancient DNA sequence have been obtained, a reference genome sequence and dense genome-wide marker data (WGS or exome capture) of a diversity panel of extant genotypes are needed to properly contextualize ancient sequences. Besides, complementary datasets such as transcriptome sequencing (RNA-Seq), epigenetic data and the inclusion of representative genome assemblies from wild and domesticated gene pools can assist in the discovery of alleles or genes related to domestication and crop improvement.

## Domestication of Legumes

### Domestication traits

Domesticated plants are often distinguished from their wild progenitors by several morphological, biochemical and developmental traits, collectively referred to as the domestication syndrome (Harlan et al. 1973, Hammer 1984). The genetic makeup of crop plants continues to evolve post domestication; hence, not all phenotypic differences between wild and domesticated forms are by necessity domestication traits. Successive genetic changes after domestication episodes are common in crop plants when they colonize new environments or adapt to new needs of their cultivators, giving rise to new phenotypes, some of them as striking as interspecific differences. The concept of ‘crucial domestication traits’ was proposed (Abbo et al. 2014) to distinguish pristine domestication traits from crop evolution (improvement-related) traits. Often, crucial domestication traits are critical for profitable cultivation and show a clear dimorphism between wild and domesticated plants. On the other hand, improvement-related traits mostly display quantitative phenotype variations among wild and domesticated gene pools (Abbo et al. 2014). Such improvement footprints are the consequence of post-domestication crop diversification. In grain legumes, pod shattering/dehiscence, seed dormancy and seed size are frequently viewed as domesticated traits. But, seed dormancy is the only trait that is determined to be a crucial domestication trait in the cool-season Near Eastern legumes (Abbo et al. 2014). A certain degree of pod shattering was observed quantitatively not only in cool-season legumes such as pea, lentil, chickpea and lupin (*Lupinus* spp.) but also in warm-season legumes including soybean, common bean and *Vigna* spp. (Parker et al. 2021b). Therefore, pod indehiscence is likely to be an improvement trait that arose after domestication. Likewise, a continuum of seed sizes is seen in wild and domesticated grain legumes of the Near East, indicating that seed size is an improvement phenotype that was shaped by farmers’ preferences after domestication (Abbo et al. 2014). Other improvement traits of grain legumes involve phenology, shape of grains, nutritional quality and taste (Vaz Patto et al. 2015).

### Domestication centers

**Fig. 2:**
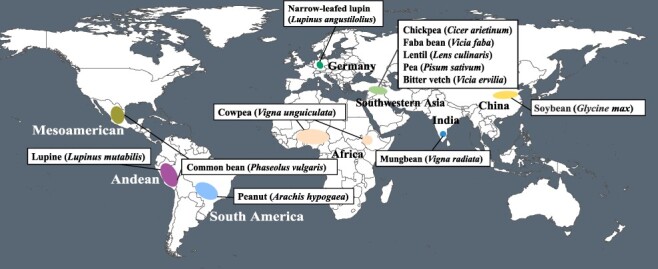
The origin and domestication of legume species. Marked regions indicate approximate locations of leguminous crop origin and domestication.

Grain legume domestication took place independently in several distinct geographical locations (**Fig. 2**), also known as ‘domestication centers’ and dispersed via human migration (exchange of people) such as faba bean or trade (exchange of ideas), e.g. in the case of common bean that was introduced to Europe from Mesoamerica (Gepts 2003, Angioi et al. 2010). Approximately 10,000 years ago, the Neolithic founder legumes including lentil, pea, chickpea and bitter vetch were domesticated in a region near the upper reaches of the Tigris and Euphrates rivers in current southeastern Turkey/northern Syria, part of the so-called Fertile Crescent (Southwest Asia) (Lev-Yadun et al. 2000, Abbo and Gopher 2017). Common bean was initially thought to originate from Northern Peru and Ecuador (South America). Recent genomic evidence supports two independent parallel domestication events (one in Mesoamerica and the other in the Andes), which both occurred between 8000 and 10,000 years ago (Gaut 2014, Rendón-Anaya et al. 2017, Singh et al. 2019). Similarly, two domestication processes were proposed for lima bean, one in Mesoamerica and one in the Andes. Application of GBS approach in wild and domesticated lima bean supported both domestication scenarios (Chacón-Sánchez and Martínez-Castilho 2017). The origin of soybean had been a matter of debate for a long time, but the development of genomic data traced its origin to the Huang-Huai Valley in Central China, the region between the Great Wall and Qinling Mountains, including both sides of the Yellow River (Han et al. 2016, Sedivy et al. 2017), where soybean was domesticated between 5000 and 9000 years ago. It is commonly held that mungbean (*Vigna radiata*), a crop with very short life cycle (70–80 days from seed to seed) originated in India 4000–6000 years ago (Fuller 2007) and then spread across Asia and Africa (Kim et al. 2015). In 1929, German breeders began to domesticate narrow-leafed lupin (*Lupinus angustifolius*) as the wild lupins have high levels of alkaloids that make their seeds toxic for direct human and livestock consumption. Intriguingly, within only 40 years’ time, narrow-leafed lupin was transformed into an established crop (Hondelmann 1984, Wang et al. 2021) and is now grown as a protein-rich feed crop in Europe and Australia.

## Charting Grain Legume Domestication by Genomics

### Genetic mapping of domestication traits (top-down approach)

#### Pod shattering.

The loss of pod dehiscence or shattering is one of the key traits that humans selected for in domesticated grain legumes. Pod shattering is imperative for seed dispersal in the wild but impedes harvest and lowers net yields under a cultivation regime. Environmental factors such as high temperatures and low relative humidity also influence pod shattering (Zhang et al. 2018). Unraveling the genetic basis of pod shattering in grain legumes has long been a major breeding and research target. To date, pod shattering is the best characterized trait associated with domesticated grain legumes (**Table 1**). Among grain legumes, pod shattering has been studied in the greatest detail in soybean. Funatsuki and his colleagues (Funatsuki et al. 2014) first cloned the major-effect Pod dehiscence 1 (*Pdh1*) gene, which encodes for a dirigent-type protein. However, the lack of genomic tools hampered the deployment of this gene in breeding to develop pod indehiscent varieties in the past. The construction of the soybean reference genome sequence (Schmutz et al. 2010) and the advent of next-generation sequencing enabled the development of a high-density genotyping assay, the SoySNP50K SNP chip (Song et al. 2013). Subsequently, trait-associated molecular markers have been identified to breed shatter-resistant soybean varieties by genomics-assisted breeding (Miranda et al. 2019). Additionally, the SHATTERING1-5 (*SHAT1-5*) gene was identified to be involved in the reduction of pod dehiscence in soybean (Dong et al. 2014). Recently, an additional gene *NST1A* (a paralog of *SHAT1-5*, also known as *NST1B*) was detected using a GWAS scan (Zhang and Singh 2020). Moreover, the shattering-related candidate gene (*Glyma09g06290*) was identified by GWAS across multiple environments, and diagnostic markers for the gene were also developed to introgress it into shattering varieties (Hu et al. 2019).

**Table 1:** Pod shattering genes in grain legumes.

Legumes	Gene/QTL	Gene product	Chromosome	Cloned?	References
Soybean	*Pdh1*	Dirigent-type protein	Gm16	Yes	(Funatsuki et al. 2014)
	*SHAT1-5*	NAC transcription factor	Gm16	Yes	(Dong et al. (2014)
	*NST1A*	NAC transcription factor	Gm07	No	(Zhang and Singh 2020)
	*Glyma09g06290*	bHLH (basic helix-loop-helix) gene family	Gm09	No	(Hu et al. 2019)
Common bean	*PvPdh1*	Dirigent-like gene	Pv03	No	(Parker et al. 2020)
	*PvMYB26*	MYB transcription factor	Pv05	No	(Rau et al. 2019)
Cowpea	*CPshat3 (VuNAC007) and CPshat5 (VuMYB26)*	NAC domain transcription factor and MYB domain protein	Vu03 and Vu05	No	(Lo et al. 2018)
Adzuki bean	*VaMYB26*	MYB domain protein	Va07	No	(Takahashi et al. 2020)
Pea	*Dpo or Dpo1*	Unknown	LG III	No	(Weeden et al. 2002)
Lentil	*Pi*	Unknown	LG IV	No	(Ladizinsky 1979)
Chickpea	*PDH1*	Dirigent-like protein	LG6	No	(Aguilar-Benitez et al. 2020)
Grass pea	*pod1 and pod2*	Unknown	LTr VII	No	(Talukdar 2011)

NAC: NAM (no apical meristem), ATAF1/2, and CUC2 (cup-shaped cotyledon).

MYB: Myeloblastosis.

In common bean (dry beans), a major QTL *PvPdh1* (ortholog of soybean *Pdh1*) associated with shattering resistance was identified (Parker et al. 2020). Subsequently, molecular markers have also been developed to help introgress it into elite varieties for reduced shattering (Parker et al. 2021a). Additionally, a large-effect QTL *PvMYB26* was mapped using a biparental population derived from a cross between the Andean snap bean cv. Midas and the wild Mesoamerican bean (G12873) (Rau et al. 2019). Recently, this locus was narrowed to a 22.5-kb region using an introgression line population and following transcriptome sequencing pinpointed the potential candidate for pod indehiscent (Di Vittori et al. 2020). Similar efforts were undertaken to identify QTL for pod shattering in other warm-season legumes, including *CPshat3* and *CPshat5* in cowpea (*Vigna unguiculata*) (Lo et al. 2018) and *VaMYB26* in adzuki bean (*Vigna angularis*) (Takahashi et al. 2020). In each case, the availability of a reference genome has facilitated the discovery of QTL and candidate genes for pod shattering. Less is known about the genetic regulation of pod shattering in cool-season legumes. In pea, *Dpo* or *Dpo1* was found to be a major factor regulating pod shattering (Ladizinsky 1979). The ortholog of *Pdh1* in soybean and common bean may be involved in pod shattering also in chickpea (Aguilar-Benitez et al. 2020). Two major mutations (*pod1* and *pod2*) associated with pod dehiscence occur in grass pea (*Lathyrus sativus*) (Talukdar 2011). Genetic mapping of pod shattering has not yet been done in the *Vicia* tribe, a research gap likely attributable to a lack of reference genomes for *Vicia* species. Very recently, reference genomes were generated for important grain legumes including pea and lentil (**Table 2**) (Kreplak et al. 2019, Ramsay et al. 2021). These new genomic resources are expected to help genetic fine mapping and cloning genes. Parallel patterns of selection have been discovered for pod shattering among grain legume species (**Table 1**) (Parker et al. 2021b). The orthologous gene of pod shattering can be searched in other grain legumes and examined for pod shattering resistance (Yundaeng et al. 2019). Such an evolutionary informed gene characterization may underpin the reduction of yield losses in other minor legumes such as tepary bean (*Phaseolus acutifolius*) and hyacinth bean (*Lablab purpureus*) that lack resources and research. Hence, a comparative genomics study at the phylogenetic level can illuminate whether other domestication traits have evolved in parallel.

**Table 2:** List of grain legumes and their genome sizes, ploidies and availability of reference genomes.

Common name	Latin name	Haploid genome size	Sequencing technologies	Assembly size (Mb)	Ploidy level	Reproduction system	References
Adzuki bean	*V. angularis*	612 Mb	Illumina + 454 XLR	443	2n = 2× = 22	Selfing	(Kang et al. 2015)
			454XLR, Pacbio	523			(Sakai et al. 2015)
Andean lupin, pearl lupin	*Lupinus mutabilis*	1 Gb			2*n* = 2× = 48	Selfing	
Bambara groundnut/ground-bean	*Vigna subterranea*	550 Mb	Illumina	512	2*n* = 2× = 22	Selfing	(Chang et al. 2019)
Beach pea/beach cowpea	*Vigna marina*	365 Mb	Illumina	365.6	2*n* = 2× = 22	Selfing	(Singh et al. 2019)
Bitter vetch	*V. ervilia*	4.2 Gb			2*n* = 2× = 14	Selfing	
Black gram	*Vigna mungo*	574 Mb	10X Genomics + HiC	499	2*n* = 2×=22	Selfing	(Pootakham et al. 2020)
Blue lupin (narrow-leafed)	*L. angustifolius*	924 Mb	Illumina	598	2*n* = 2× = 40	Selfing	(Yang et al. 2013)
			Illumina	609			(Hane et al. 2017)
			Pacbio	615.7			(Wang et al. 2021)
Chickpea	*C. arietinum* (cultivated)	740 Mb	Illumina	532.3	2*n* = 2× = 16	Selfing	(Varshney et al. 2013)
			Illumina + 454 XLR	519.8			(Jain et al. 2013)
			Illumina + 454 XLR	510.9			(Parween et al. 2015)
	*C. reticulatum* (wild)		Illumina	416			(Gupta et al. 2016)
Common bean/kidney beans/dry beans	*P. vulgaris*	587 Mb	454 XLR + Illumina + Sanger	474.3	2*n* = 2× = 22	Selfing	(Schmutz et al. 2014)
			454 XLR + SOLID + Sanger	549.6			(Vlasova et al. 2016)
Common vetch	*Vicia sativa*	1.8 Gb	Illumina + Pacbio	1541	2*n* = 2× = 14	Selfing	(Shirasawa et al. 2021)
			ONT PromethION + HiC	1651			(Xi et al. 2022)
Cowpea/yardlong bean	*V. unguiculata*	613 Mb	Illumina	568–610	2*n* = 2× = 22	Selfing	(Spriggs et al. 2018)
			Pacbio + BioNano	519.4			(Lonardi et al. 2019)
			Illumina	632.8			(Xia et al. 2019)
Faba beans/broad bean	*V. faba*	13 Gb			2*n* = 2× = 12	Mixed mating	
Grass pea	*L. sativus*	6.2 Gb	ONT PromethION	6237	2*n* = 2× = 14	Mixed mating	(Emmrich et al. 2020)
Horse gram	*Macrotyloma uniflorum*	343.6 Mb	Illumina	259.2	2*n* = 2× = 20	Selfing	(Shirasawa et al. 2021)
Hyacinth bean	*L. purpureus*	367 Mb	Illumina	395	2*n* = 2× = 22	Selfing	(Chang et al. 2019)
Jack bean	*Canavalia gladiata*				2*n* = 2× =22	Mixed mating	
Lentil	*L. culinaris* (cultivated)	4 Gb	Pacbio + ONT PromethION + BioNano+ HiC	3760	2*n* = 2× = 14	Selfing	(Ramsay et al. 2021)
	*Lens ervoides* (wild)			2870			
Lima bean	*Phaseolus lunatus*	622 Mb	Illumina	623	2*n* =2× = 22	Selfing	(Garcia et al. 2021)
Moth bean	*Vigna aconitifolia*	1.1 Gb			2*n* = 2× = 22	Selfing	
Mungbean/Green gram	*V. radiata*	579 Mb	Illumina + 454 XLR	431	2*n* = 2× = 22	Selfing	(Kang et al. 2014)
			Pacbio	475			(Ha et al. 2021)
Pea/dry pea	*P. sativum*	4.4 Gb	Illumina + Pacbio + BioNano	3920	2*n* = 2× = 14	Selfing	(Kreplak et al. 2019)
Peanut	*Arachis duranensis*	1.1 Gb	Illumina	1025	2*n* = 2× = 20	Selfing	(Bertioli et al. 2016)
			Illumina	1051			(Chen et al. 2016)
	*Arachis ipaensis*	1.4 Gb	Illumina	1338	2*n* = 2× = 20	Selfing	(Bertioli et al. 2016)
			Illumina	1391			(Lu et al. 2018)
	*A. hypogaea* (cultivated)	2.7 Gb	Pacbio + HiC	2,556.30	2*n* = 4× = 40	Selfing	(Bertioli et al. 2016)
			Pacbio + HiC	2506			(Zhuang et al. 2019)
			10X Genomics + Pacbio+ BioNano	2552			(Chen et al. 2019)
	*Arachis monticola* (wild)		Pacbio + BioNano + HiC	2620			(Yin et al. 2018)
Pigeonpea	*Cajanus cajan*	852 Mb	Illumina	605.7	2*n* = 2× = 22	Mixed mating	(Varshney et al. 2012)
			Illumina	648.2			(Mahato et al. 2018)
			None	548			(Marla et al. 2020)
Rice bean	*V. umbellata*	562 Mb	Illumina + Pacbio	414	2*n* = 2× =22	Selfing	(Kaul et al. 2019)
Soybean	*G. max* (cultivated)	1.1 Gb	Sanger	1115	2*n* = 2× = 40	Selfing	(Schmutz et al. 2010)
	*Glycine soja* (wild)		Illumina	813–985			(Li et al. 2014)
	*G. soja* (wild)		Illumina	868			(Qi et al. 2014)
	*G. max* (cultivated)		Pacbio + BioNano + HiC	1025			(Shen et al. 2018)
	*G. soja* (wild)		Pacbio + BioNano+ HiC	1,013.20			(Xie et al. 2019)
			Pacbio + BioNano + HiC	992.3–1,059.8			(Y. Liu et al. 2020)
	*Glycine latifolia* (wild)		10X Genomics	939.3			(Liu et al. 2018)
Tepary bean	*P. acutifolius*	682 Mb	Pacbio + HiC	512.6–661.8	2*n* = 2× = 22	Selfing	(Moghaddam et al. 2021)
White lupin	*Lupinus albus*	451 Mb	Pacbio + BioNano	451	2*n* = 2× = 50	Selfing	(Hufnagel B et al. 2020)
			Pacbio + HiC	558.9			(Xu et al. 2020)
Winged beans	*Psophocarpus tetragonolobus*	1.2 Gb			2*n* = 2× = 18	Selfing	
Yellow lupin	*L. luteus*				2*n* = 2× = 52	Selfing	

#### Seed dormancy.

Seed dormancy is a crucial domestication trait in certain crop plants. In the wild, dormant seeds delay germination and promote survival and fitness via the buildup of soil seed banks (Finch-Savage and Footitt 2017). Seed dormancy in a domesticated crop may cause asynchronous germination, lower crop performance and ultimately, severely limits yields (Abbo et al. 2008, 2011). The physiological mechanisms and the molecular genetics behind seed dormancy are not well-studied in grain legumes. Physical dormancy, namely impermeable seed coats, seems to play a bigger role than physiological dormancy, i.e. inhibition of embryo development (Martin 1946). Major-effect loci controlling seed dormancy were identified in lentil (Ladizinsky 1985), blue lupine (*L. angustifolius*) (Forbes and Wells 1968), cowpea (Kongjaimun et al. 2012), rice bean (*Vigna umbellata*) (Isemura et al. 2010) and mungbean (*V. radiata*) (Isemura et al. 2012). In pea and common vetch, two QTLs were mapped for seed dormancy (Donnelly et al. 1972, Weeden 2007). Nonetheless, these studies have used a limited number of molecular markers from low-throughput and polymerase chain reaction-based simple-sequence repeats that restrict fine mapping to narrow genetic intervals and subsequent causal gene discovery. But in common bean, the development of reference genome and resequencing data allowed mapping the seed dormancy locus within a 118-kb genomic interval with a potential candidate gene (Soltani et al. 2021). The first cloned seed dormancy gene in legumes was *GmHs1-1*, a soybean gene that encodes a calcineurin-like metallophosphoesterase transmembrane protein (Sun et al. 2015). This achievement was facilitated by sequencing data from a mapping population that permit to fine map to a 22-kb region harboring two genes (Sun et al. 2015). Furthermore, with the aid of high-throughput genomic data, the green seed coat gene, also known as *G allele*, which is also responsible for seed dormancy was cloned, and this gene was found to have undergone parallel selection in different crop families (Wang et al. 2018). Likewise, the resequencing approach has identified two candidate genomic regions spanning 2.4 Mb and 0.74 Mb associated with seed dormancy in peanut (Kumar et al. 2019). The genetic linkage or co-localization of QTL controlling seed dormancy and pod shattering was also reported in lentil (Ladizinsky 1985) and common bean (Soltani et al. 2021), indicating that pleiotropy or tight linkage of several loci could be an important attribute of domestication (Harlan et al. 1973, Meyer and Purugganan 2013).

### Genome scans for footprints of selection (bottom-up approach)

QTL scans aim at identifying genomic regions controlling traits hypothesized by researchers to be involved in the domestication syndrome. By contrast, bottom-up approaches are hypothesis-free in that they do not require predictions as to which phenotypes were selected for. While selection scans are phenotype-agnostic, the co-location of sweeps and QTL for domestication traits reinforces the confidence in either of them.

In soybean, a strong selective sweep spanning approximately 116 kb was spotted at the *SHAT1-5* locus: all soybean landraces shared a single haplotype, highly indicative of strong recent selection (Dong et al. 2014). A similar signature was observed in 100 kb surrounding the *PvPdh1* locus in common bean (Parker et al. 2021a). For seed dormancy, a 160-kb selective sweep covering the seed dormancy gene *GmHs1-1* was detected in soybean using resequencing data (Sun et al. 2015). Furthermore, resequencing data from a panel of 302 accessions comprising wild germplasm, landraces and improved cultivars demonstrated domestication selection signals as well as post-domestication improvement traits including seed weight, seed coat color and oil content in soybean (Zhou et al. 2015). Zhou et al. (2015) pointed out that sweep scans can refine prior QTL mapping results. For instance, previous studies (Liu et al. 2007) had identified a pod shattering QTL spanning 12 Mb, while the selection scan of wild soybeans versus landraces narrowed down the interval to a 190-kb region containing only 14 genes. Refined intervals can guide the prioritization, validation and subsequent functional characterization of domestication genes. As in QTL mapping, the resolution of sweep scans is determined, among other things, by genome size, sequence diversity and recombination rates. Different contrasts in sweep scans (e.g. wild progenitor vs. landraces and landraces vs. elite cultivars) may give clues about the relative timing of selection. For example, Zhou et al. (2015) showed that seed size was targeted during the domestication process, while seed color was likely shaped by post-domestication variety selection. Selective sweeps can also tag regions involved in traits that are adaptive in both wild and domesticated forms but subject to different selective regimes in either taxon. For instance, Varshney et al. (2019) identified the selection signals possibly related to key biotic and abiotic stress tolerance in resequencing of 429 diverse chickpea accessions (Varshney et al. 2019).

## Effect of Domestication on Secondary Metabolites

Grain legumes are not only an important source of proteins in the human diet, but they are also rich in secondary metabolites such as polyphenols, alkaloids and saponins (Gupta 1987). In the wild, these play a role in defense against herbivores and pathogens. In the domesticated form, secondary metabolites may either promote human health or act as anti-nutritional factors. Domestication and post-domestication improvement have altered contents of the essential primary and secondary metabolites in legumes. Understanding the underlying genetic factors will boost breeding and inform metabolic engineering to improve the nutritional value of current grain legumes. Genes involved in biosynthetic pathways for health-promoting metabolites that were lost in the domestication bottleneck may be re-introduced from extant wild relatives.

### Beneficial compounds

Bioactive compounds in legumes may have beneficial effects in the prevention and treatment of chronic diseases in human such as cardiovascular ailments, diabetes, cancer and neurogenerative diseases (Guaadaoui et al. 2020). For example, legumes produce 3,4-dihydroxyphenyl-L-alanine, which is used in the treatment of Parkinson’s disease (Etemadi et al. 2018). An examination of wild and domesticated accessions supported a selection pressure for higher tryptophan levels in chickpea (Kerem et al. 2007). Consistent with intense breeding for high oil content in soybean, genes regulating fatty acid biosynthesis showed footprints of selection in soybean when compared to the wild progenitors (Zhou et al. 2015) and several genes involved in fatty acid biosynthesis were co-located with oil content QTLs (Iqbal et al. 2020). Carotenoids are considered as antioxidants in food systems. A study that investigated the 10 most important grain legumes [peanut, chickpea, soybean, vetch, lentil, lupin, common bean, pea, faba bean (unknown progenitor) and cowpea] and their wild relatives have found, on average, 48% lower carotenoid content, including lutein and zeaxanthin in domesticated legumes (Fernández-Marín et al. 2014). Protein quality is determined by the essential amino acid composition, digestibility and bioavailability of individual amino acids (Vaz Patto 2016). High-quality protein legumes may play an important role in a future human diet less reliant on carbon-intensive meat production (Poore and Nemecek 2018). However, little is known about the genetic basis of protein quality (in terms of improved amino acid composition and digestibility) in grain legumes. A large-scale screening of genetic resources is needed to identify natural variation for protein quality and breed for improved protein content and quality. Genotyping of large germplasm collections has become feasible with contemporary technology like GBS or skim sequencing (Milner et al. 2019). Therefore, efforts should be made to fingerprint diverse gene pools and explore variation for domestication and improvement-related traits to meet current nutritional requirements for human beings and livestock.

### Anti-nutritional compounds

Some of the secondary metabolites in grain legumes cause indigestibility, making them unpalatable to humans and animals. Such secondary metabolites are known as anti-nutritional factors. Examples are tannins, vicine and convicine (Kumar et al. 2021). The color of the seed coat and seed tissues is primarily controlled by secondary metabolites including beneficial carotenoids, anthocyanin and antinutrient compounds—tannins (Espinosa-Alonso et al. 2006). Changes in seed coat color are commonly observed in domesticated and improved legumes as compared to their wild progenitors. For instance, the wild common beans have colored seed coats, while elite varieties are predominantly black seeded (McClean et al. 2018). A survey of thousands of wild and domesticated soybeans illustrated that all wild soybeans have black seed coats whereas the domesticated soybeans have a range of seed coat colors including yellow, brown and green (Jeong et al. 2019), but the modern cultivars are predominantly yellow (also called colorless or white). For many decades, selective breeding efforts have targeted to eliminate anti-nutritional factors. The bitter taste caused by alkaloids has been intentionally bred out of legumes. In yellow lupin (*Lupinus luteus*), the domesticated cultivar has much lower alkaloid content than the wild form (Iqbal et al. 2020). Similarly, faba bean breeding has largely focused on the development of anti-nutrition-free (such as tannins and low vicine and convicine) cultivars. With an integrative analysis of transcriptomes and metabolomes, the key regulatory gene involved in vicine and convicine pathway was disclosed (Björnsdotter et al. 2021). Phytic acid (myo-inositol-1,2,3,4,5,6-hexakisphosphate) is the major storage form of phosphorus in grain legumes such as soybean and common bean. Its poor digestibility and chelation of mineral cations (such as iron, zinc, potassium, calcium and magnesium) reduce the nutritional availability (Erdman 1981). The early development of genome sequence and adoption of WGS had located the low-phytate genes in soybean (Maroof et al. 2009). Raffinose family oligosaccharides, which cause flatulence in humans and animals, are also abundant in the legume family (Elango et al. 2022). Genomic approaches have also facilitated the development of molecular markers associated with low-raffinose phenotype in soybean (Dierking and Bilyeu 2008). The identification of key genes associated with diverse secondary metabolite pathways is therefore imperative to optimize the target metabolite content in commercial cultivars. The availability of genomic resources accelerates such quality improvements in seed legumes. Yet, the reduction in secondary metabolites in domesticated cultivars might make them susceptible to biotic stresses compared to their wild counterparts. It is also equally important to identify resistance alleles in the exotic material and stack them in modern cultivars.

## Effect of Domestication on Symbiosis

Legumes are capable of establishing a root-nodule symbiosis with nitrogen-fixing soil bacteria called rhizobia, which greatly increases nitrogen availability for the plant host. This symbiosis plays a crucial role in developing sustainable farming systems. However, domestication and intense artificial selection for high yield with the use of chemical fertilizers may have disrupted the plant–microbe interaction, reducing the rate of bacterial nitrogen fixation or nitrogen uptake by the host (Porter and Sachs 2020, J. Liu et al. 2020). Plant cultivation often includes the use of manure or artificial fertilizers, so that domesticated forms might have become dependent on readily available soil nitrogen (Porter and Sachs 2020). The ancestral symbiosis has been retained but the host’s ability to favor the most efficient bacterial partners might have been compromised (Porter and Sachs 2020). A few small-scale studies have explored the effect of domestication and crop evolution on symbiosis. The investigation of host-range evolution using 80 isolates showed that only 34% of the strains were able to nodulate cultivated legume species while 89% were able to nodulate the other wild species, demonstrating that the domesticated species are loosening their interaction with beneficial bacteria as compared with wild legumes (Mutch and Young 2004). It has been observed that the wild soybean (*Glycine soja*) can recruit more diverse *Bradyrhizobium* strains in its rhizosphere than the cultivated soybean (Chang et al. 2019). Similarly, a nodulation experiment in chickpea indicated that the wild progenitor of chickpea (*Cicer reticulatum*) entered symbiotic relationships with more diverse *Mesorhizobium* than domesticated chickpea (Kim et al. 2014). This was ascribed to the loss of alleles of nod factor signaling genes under domestication in chickpea (Kim et al. 2014). In contrast, Greenlon et al. (2019) showed that the wild chickpeas had evolved symbiosis with specific bacteria whereas the domesticated chickpea exhibited symbiosis with diverse bacterial strains as they spread to new locations, indicating the co-evolution of rhizobia with chickpea domestication and diversification (Greenlon et al. 2019). Hence, the symbiosis might vary by crop and geography. Genomic approaches should be employed to elucidate pathways and genes associated with legume-rhizobial symbiosis in wild legumes to improve biological nitrogen fixation in domesticated legumes for sustainable agriculture. Furthermore, the latest advancement in sequencing has enabled large-scale metagenomics to understand plant–microbe interactions (Knief 2014).

## Conclusion and Future Perspectives

Replacing meat-based protein diets with plant-based protein diets is critical to maintain the global food and nutritional sustainability (Shepon et al. 2018). One aspect of this transition is the increased local production of protein-rich leguminous crops. In that respect, a better understanding of legume domestication and diversification is indispensable. The biological differences between legumes and cereals may have influenced the selection of potential plants by the first farmers (Abbo et al. 2009). Substantial biological differences were spotted between legumes and cereals including seed dormancy, seed dispersal and population structure, suggesting that hunter-gatherers interacted differently with wild legumes than with wild cereals (Abbo et al. 2009, 2009). Deeper insights on cereal and legume domestication will allow us to understand the biological as well as cultural processes associated with domestication and evolution. Additionally, such knowledge promotes crop improvements not only in cereals but also in grain legumes, which is important for achieving a resilient food system. However, to date, a comprehensive study that compares the domestication syndromes between cereal and legumes at a genome-wide level and with single-gene resolution is still missing. We attribute this situation to the lack of investment/research effort or genomic resources available for important grain legumes as compared to important cereals (maize, wheat and barley), all of which have had their genome sequenced in the last decade. For example, grain legumes had received far less research funding from the United States Department of Agriculture between 2008 and 2019 (Bollington et al. 2021). In parallel, the greater investment in major cereals and the development of high-throughput genomics resources enabled innovative solutions to increase crop yield and performance under different biotic and abiotic conditions (Varshney et al. 2021).

In legumes, it has been observed that disease-resistance-related genes were lost as a result of domestication and subsequent crop improvement (Zhou et al. 2015, Varshney et al. 2019). This implies that a reference genome from a single domesticated crop cannot capture all the resistance-conferring alleles and might cause reference bias in estimating genetic variation between wild and domesticated populations. The construction of a pan-genome (the universe of DNA sequences within a species) is advantageous to capture diverse alleles (Jayakodi et al. 2021). The cost reduction and recent development in accurate long-read sequencing technologies have simplified the genome assembly process in several crops (Jayakodi et al. 2021). Moreover, building a pan-genome with the inclusion of domesticated and wild species (i.e. primary gene pool) of a grain legume is profitable as it may capture genes or alleles for disease resistance, legume-rhizobial symbiosis and nutritional quality. Furthermore, pan-genome studies in crop plants demonstrated that past geographic range expansion and recent breeding have been accompanied by large structural variations such as inversions and presence/absence variants (Jayakodi et al. 2020, Hufford et al. 2021). In this vein, the pan-genome will become an important tool for grain legume research in order to discover resistance and adaptation genes in historic germplasm and deploy them in newly developed cultivars. Until now, among legumes, pan-genomes with chromosome-level assemblies have been constructed only for soybean (Y. Liu et al. 2020). Likewise, epigenetic variation, e.g., changes such as changes in DNA methylation or histone methylation cause phenotypic variations during plant evolution (Ding and Chen 2018). To date, comprehensive studies focusing on epigenetic changes associated with domestication are lacking in legumes. Very few efforts have been made to study the epigenetic changes in relation to abiotic stresses (Varotto et al. 2020). Like other research areas, the analysis of ancient DNA in legumes is still in its incipient stage. The discovery of 14,000-year-old faba bean-like seeds in the prehistoric site of el-Wad Terrace, Mount Carmel, Israel (Caracuta et al. 2016), suggests a Levantine origin of that crop. However, the charred state of seeds limits the application of genomics in ancient remains.

Lost beneficial alleles can be brought back from the wild to domesticated accessions via introgression. However, this method has been hampered by linkage drag (co-transfer of undesirable alleles or deleterious alleles). Nonetheless, this problem can be alleviated by deploying molecular maps and utilizing advanced backcross QTL approach with the aid of genomic tools. Pratap et al. (2021) summarized the successful examples of introgression breeding in legumes (Pratap et al. 2021). Nevertheless, the potential of introgression breeding in legumes needs to be better exploited to transfer beneficial genes from the wild to develop superior cultivars. Re-domestication, on the other hand, revives the cultivation of lost species. For instance, the Marama bean (*Tylosema esculentum* (Burchell) A. Schreiber) and the African locust bean (*Parkia biglobosa* (Jacq.) R.Br. ex G.Don) have been targeted for domestication with the use of genomes (Smýkal et al. 2018). Modern genome-editing technologies such as Clustered Regularly Interspaced Short Palindromic Repeat/ CRISPR associated protein 9 have been shown to be a reliable tool for the rapid and accurate improvement of target traits in different plants (Zaidi et al. 2020). However, the optimization of an efficient transformation system in legumes is the major bottleneck due to genotype dependence and recalcitrance. Still, the success rate is inadequate in important grain legumes such as pea, chickpea, pigeonpea and mungbean (Choudhury and Rajam 2021). It is anticipated that the rapid methodological advancements in functional genomics might alleviate the transformation bottleneck in the future. Thus, a combination of genomics and genome editing offers a unique opportunity to understand crop domestication and evolution and translate the acquired knowledge into improved grain legume varieties.

## Data Availability

No new data were generated in support of this review article.
